# A minimal cellulosome‐like system in *Cellulosilyticum lentocellum*

**DOI:** 10.1002/2211-5463.70330

**Published:** 2026-08-19

**Authors:** John Allan, Amias Alstrom‐Moore, Gary W Black

**Affiliations:** ^1^ Department of Applied Sciences, Health and Life Sciences Ellison Building, Northumbria University Newcastle upon Tyne UK

**Keywords:** cellulase, cellulosome, cohesin, dockerin, enzymology

## Abstract

Cellulosomes are efficient enzymatic nanomachines which have arisen for the degradation of cellulosic biomass. They are found abundantly in soil‐dwelling microbes and bacteria which thrive in the stomachs of ruminant mammals. Two protein domains, cohesins and dockerins, characterise cellulosomes. These domains interact with each other to form, in many cases, enormous complexes with as many as 160 individual proteins. However, genome annotation of *Cellulosilyticum lentocellum DSM 5427* revealed a single cohesin domain (encoded by Clole_2599) and a single dockerin domain (Clole_2598). Therefore, we recombinantly expressed ClcC and ClcD and found they form a (predicted ~ 104 kDa) heterodimeric complex. We show that this complex formation enhances cellulase activity approximately 2‐fold on insoluble microcrystalline cellulose and 1.25‐fold on soluble carboxymethyl cellulose. Moreover, we identified two additional candidate interacting partners for ClcC, one of which appears to bind via a non‐canonical interface. These findings suggest that cellulosomal principles can operate in highly reduced cohesin‐dockerin systems.

## Graphical abstract

Cellulose‐degrading bacteria typically use cellulosomes, large multi‐enzyme complexes on a scaffold protein. In *Cellulosilyticum lentocellum,* we characterise a far smaller arrangement, a single scaffold bound to one cellulase through a single cohesin‐dockerin interaction. The scaffold's cellulose‐binding module anchors the pair to substrate and raises the enzyme's activity on insoluble cellulose. We argue this represents a minimal cellulosome.

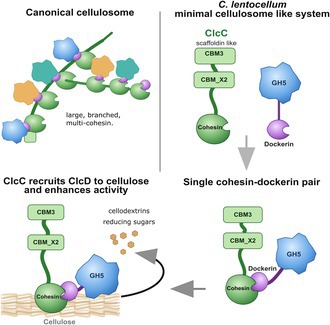

**Abbreviations**
BIg‐7: bacterial immunoglobulin‐like domain type VII
CAZyme: carbohydrate‐active enzyme
CBM: carbohydrate‐binding module
CDI: calcium dilution and incubation
CFE: cell‐free extract
CMC: carboxymethyl cellulose
cpTM: chain‐pair predicted template modelling score
DNS: 3; 5‐dinitrosalicylic acid
DP: degree of polymerisation
GEBA: Genomic Encyclopedia of Bacteria and Archaea
GH: glycoside hydrolase
HPAEC‐PAD: high‐performance anion‐exchange chromatography with pulsed amperometric detection
ipTM: inter‐chain predicted template modelling score
MCC: microcrystalline cellulose
PAE: predicted aligned error
PASC: phosphoric acid‐swollen cellulose
PCSb: peptone cellulose solution B
pLDDT: predicted local distance difference test
pTM: predicted template modelling score
SLH: S‐layer homology

Cellulosomes are complex enzymatic apparatuses secreted and, in some cases, anchored to cell surfaces for the purpose of cellulose and hemicellulose degradation to simple sugars that can be utilised by bacterial cells [1]. These molecular machines are assembled through near‐covalent interactions between cohesin domains on scaffoldin proteins and dockerin domains on catalytic enzymes, with cohesin‐dockerin interactions typically being species‐specific [2, 3]. This modular architecture facilitates the assembly of complexes containing as many as 160 proteins [3], with cellulases and xylanases comprising the typical enzymatic repertoire, though other enzymes have been described [4]. Cellulosomes may be either cell surface‐anchored via scaffoldin‐associated domains such as SLH modules, or secreted as cell‐free scaffoldins lacking these anchoring domains. Cell‐free scaffoldins have been observed in species including *Acetivibrio thermocellus* (previously *Clostridium thermocellum*), *Acetivibrio clariflavus* (*previously Clostridium clariflavum*) and *Acetivibrio cellulolyticus* and are thought to facilitate degradation of remote cellulosic substrates [4].

Cellulosome architecture exists along a spectrum of complexity. The canonical cellulosome is produced by *A. thermocellus* [3], representing one of the most elaborate systems characterised to date. In this system, a cell surface‐anchored scaffoldin recruits cellulases and xylanases bearing dockerin domains, as well as additional dockerin‐containing scaffoldin proteins (termed type II scaffoldins), giving rise to large, highly branched structures [5]. At the opposite end of this spectrum, a variety of well‐characterised simpler cellulosomes consist of a single primary scaffoldin and a handful of dockerin modules [6]. For example, the primary scaffoldin of *A. cellulolyticus* contains 7 cohesins, *Ruminococcus flavefaciens* possesses 21 cohesins distributed across multiple scaffoldins, and *Pseudobacteroides cellulosolvens* has 78 cohesins across 31 different scaffoldins [6].

The simplest system described to date belongs to *Clostridium saccharoperbutylacetonicum*, which has as few as eight dockerins and two cohesin modules [6]. Given the diverse ecological niches occupied by cellulosome‐producing organisms and the metabolic burden of synthesising and maintaining large multi‐protein complexes, it is surprising that no smaller cellulosomes have been characterised.

*Cellulosilyticum lentocellum*, formerly *Clostridium lentocellum*, is closely related to many cellulosome‐utilising organisms [7, 8, 9] and was first isolated in 1985 from estuarine sediment exposed to paper‐mill runoff and residential wastewater. Despite some exploration of its potential utility [10, 11], there has been surprisingly little work focused on the mechanism behind the organism's cellulolytic capabilities. Genomic analysis revealed a predicted two‐gene operon (Clole_2599 and Clole_2598, hereafter clcC and clcD) encoding a single cohesin domain and a single dockerin‐bearing enzyme, a markedly reduced arrangement compared with canonical enzyme cellulosomes [8]. Given their co‐localisation and the canonical pairing of cohesin‐dockerin modules, we set out to determine whether ClcC and ClcD function together as a minimal cellulose‐targeting complex, and whether additional *C. lentocellum* proteins, despite lacking canonical dockerins, interact with the ClcC cohesin.

## Results and Discussion

### Sequence relationships and domain architecture of ClcC and ClcD

The original genome annotation of *C. lentocellum* DSM 5427 predicted thirteen cellulose‐degradation enzymes [8]. We annotated the genome for carbohydrate‐active enzymes and examined those predicted to act on cellulose (Table 1). The enzymes predicted to act on cellulose comprise endocellulases (GH9, GH5, GH74) and a GH48 cellobiohydrolase, most carrying a predicted signal peptide and several also carrying one or more fused cellulose‐binding modules. A second group (GH1/GH3 β‐glucosidases and GH94 cellobiose/cellodextrin phosphorylases) is predicted to act on the resulting cellobiose and cello‐oligosaccharides. ClcD is annotated as one of several cellulases lacking a fused CBM but the only one bearing a dockerin, while ClcC is the only cohesin‐bearing protein (Table 1).

**Table 1:** Predicted cellulose‐degradation enzymes of *Cellulosilyticum lentocellum* DSM 5427 (GenBank CP002582.1). Carbohydrate‐active enzymes were annotated with dbCAN3 (consensus of ≥ 2 of 3 tools) and secretion predicted with SignalP 6.0. Enzymes are grouped by the substrate on which they act; Enzyme Commission (E.C.) numbers are dbCAN_sub predictions and “–” indicates none assigned.

Locus tag	Predicted E.C.	Annotation	CAZy family	Signal peptide	CBMs	Localisation
**Cellulose**						
Clole_0675	–	Glycoside hydrolase family 9 protein	CBM4 + GH9	Sec/SPI	CBM4	Secreted/cell‐associated
Clole_1765	–	Glycoside hydrolase family 5 protein	CBM65 + GH5_4 + CBM65 + CBM2 + CBM2	Sec/SPI	CBM2 + CBM65	Secreted/cell‐associated
Clole_3668	3.2.1.4|3.2.1.151|3.2.1.176|3.2.1.‐	Glycoside hydrolase family 48 protein	GH48	Sec/SPI	–	Secreted/cell‐associated
Clole_2598	3.2.1.4	**Glycoside hydrolase family 5 protein (ClcD)**	GH5_1	Sec/SPI	–	Secreted/cell‐associated
Clole_1921	–	Glycoside hydrolase family 5 protein	GH5_2	Lipoprotein/SPII	–	Secreted/cell‐associated
Clole_3976	–	Glycoside hydrolase family 5 protein	GH5_2	Lipoprotein/SPII	–	Secreted/cell‐associated
Clole_2863	–	Glycoside hydrolase family 5 protein	GH5_4 + CBM2	Sec/SPI	CBM2	Secreted/cell‐associated
Clole_1968	–	Glycoside hydrolase family 5 protein	GH5_55 + CBM32 + CBM3	Sec/SPI	CBM3 + CBM32	Secreted/cell‐associated
Clole_2677	–	Glycoside hydrolase family 74 protein	GH74 + GH74	None	–	Cytoplasmic
Clole_3660	3.2.1.4|3.2.1.151	Glycoside hydrolase family 74 protein	GH74 + GH74 + CBM46	Sec/SPI	CBM46	Secreted/cell‐associated
Clole_0038	–	Glycoside hydrolase family 9 protein	GH9	Lipoprotein/SPII	–	Secreted/cell‐associated
Clole_0683	–	Glycoside hydrolase family 9 protein	GH9	Sec/SPI	–	Secreted/cell‐associated
Clole_0485	3.2.1.4|3.2.1.73|3.2.1.176|3.2.1.78|3.2.1.151|3.2.1.‐	Glycoside hydrolase family 9 protein	GH9 + CBM3	None	CBM3	Cytoplasmic
Clole_0709	3.2.1.4	Glycoside hydrolase family 9 protein	GH9 + CBM3 + CBM3	Sec/SPI	CBM3	Secreted/cell‐associated
Clole_3667	3.2.1.4|3.2.1.73|3.2.1.176|3.2.1.78|3.2.1.151|3.2.1.‐	Glycoside hydrolase family 9 protein	GH9 + CBM3 + CBM3	Sec/SPI	CBM3	Secreted/cell‐associated
**Cellobiose/cello‐oligosaccharides**						
Clole_3150	–	β‐glucosidase	GH1	None	–	Cytoplasmic
Clole_3922	3.2.1.21|3.2.1.86	β‐glucosidase	GH1	None	–	Cytoplasmic
Clole_0676	–	β‐glucosidase	GH3	None	–	Cytoplasmic
Clole_1639	–	β‐glucosidase	GH3	None	–	Cytoplasmic
Clole_2843	–	β‐glucosidase	GH3	Lipoprotein/SPII	–	Secreted/cell‐associated
Clole_2576	2.4.1.20	Cellobiose/cellodextrin phosphorylase	GH94	None	–	Cytoplasmic
Clole_3233	–	Cellobiose/cellodextrin phosphorylase	GH94	None	–	Cytoplasmic
Clole_3988	2.4.1.31	Cellobiose/cellodextrin phosphorylase	GH94	None	–	Cytoplasmic
Clole_3989	–	Cellobiose/cellodextrin phosphorylase	GH94	None	–	Cytoplasmic

Domain architectures were predicted using SMART [12] (Fig. S5). The two genes lie ~ 40 bp apart, downstream of a single predicted promoter, with a separate ribosome‐binding site predicted upstream of each (Fig. S5A). This suggests they are co‐transcribed as a single operon but carry independent translation signals. The gene locus Clole_2599 (*clcC*) encodes the scaffoldin‐like protein ClcC, which carries an N‐terminal CBM3, an intervening CBM_X2 (carbohydrate‐binding domain), and a C‐terminal cohesin domain. The CBM3 domain is a common feature of cellulosomal scaffoldin proteins, typically found on ScaA orthologues, and facilitates close interaction with cellulose substrate chains. However, ClcC does not seem to possess the classical SLH modules associated with cell surface binding consistent with cell‐free cellulosome architectures [4].

The CBM_X2 domain is an immunoglobulin‐like module that has been characterised as having cellulose‐binding capability and is commonly found in bacterial cellulases [13, 14]. Moreover, X2 domains are thought to influence the surface localisation of simple cellulosomes, with evidence suggesting they may modulate interactions with cellulosic substrates or other cell surface components [4].

The gene locus Clole_2598 (*clcD*) encodes the enzyme ClcD, which bears an N‐terminal GH5 cellulase domain (residues 45 to 387) and a C‐terminal dockerin (residues 449 to 518). Based on UniProt sequences, ClcC and ClcD have predicted molecular masses of ~ 45.9 kDa (F2JID6) and ~ 57.8 kDa (F2JID5), respectively. Notably, these are the only annotated cohesin and dockerin domains in the genome, an unusual feature for a cellulosome‐producing organism [15]. As the cohesin‐dockerin assembly is the defining feature of cellulosomal architecture [16], the arrangement of this pairing is consistent with cellulosomal function, albeit in a highly reduced form.

clustalw alignments revealed that the ClcD dockerin shares 57% identity with the *A. thermocellus* XynB dockerin and 59.65% identity with the *A. clariflavus* GH9 dockerin; in phylogenetic analysis, it clustered closely with these thermophilic clostridial dockerins (Fig. S2). However, the conserved hydroxyl pair (Ser/Thr or Ser/Ser) that mediates cohesin recognition is retained in only one of the two dockerin repeats – present as Ser‐Thr in the N‐terminal repeat but replaced by Glu‐Gly in the C‐terminal repeat (Fig. S1A). Because the dual binding mode depends on this pair being conserved in both repeats [17], ClcD is unlikely to bind in the dual mode and instead presents a single cohesin‐recognition interface [16]. In contrast, the ClcC cohesin appears phylogenetically distinct: although it shares 98% identity with a cohesin from *Cellulosilyticum* sp. (Fig. S1), unrooted trees place it in a separate clade from canonical scaffoldin cohesins (Fig. S3).

This combination of a canonical dockerin with a phylogenetically divergent cohesin is unusual and may reflect the ecological context of this minimal system. Intriguingly, the WCF‐2 genome, which encodes the closest ClcC cohesin homologue, lacks any identifiable dockerin‐encoding genes [18]. Cross‐species cohesin‐dockerin interactions have been documented, particularly among type II cohesins where broad interspecies recognition is common [19], and such cross‐species interactions may provide benefits under resource‐limited conditions by enabling degradation without the metabolic cost of maintaining a complete cellulosome [4].

### Biochemical characterisation of ClcC and ClcD

**Fig. 1:**
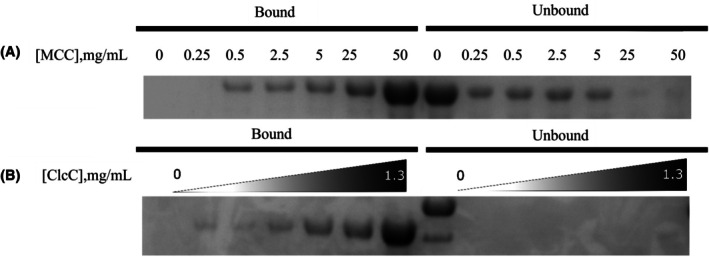
ClcC binding to microcrystalline cellulose (MCC). (A) ClcC (1.36 mg·mL^−1^) was incubated with MCC (0–50 mg·mL^−1^) for 3 h at 4 °C with rotation in CDI buffer. After centrifugation, supernatant (unbound) and pellet (bound) fractions were collected and analysed by sodium dodecyl sulfate polyacrylamide gel electrophoresis (SDS/PAGE). (B) MCC was fixed at 25 mg·mL^−1^ and ClcC was titrated from 1.36 to 0.0425 mg·mL^−1^ for 3 h at 4 °C; fractions were processed as in (A). Bands correspond to ClcC, predicted molecular mass 45.9 kDa (UniProt F2JID6). In (B), the marker lane shows molecular weight standards of ~ 50 kDa (lower band) and ~ 70 kDa (upper band).

To characterise this putative cellulosomal operon, we first wanted to test if ClcC binds cellulose via its CBM3 domain. We expressed ClcD and ClcC heterologously in *E. coli* BL21(DE3) and purified them by immobilised metal affinity chromatography. ClcC bound strongly to microcrystalline cellulose. SDS/PAGE analysis showed that ClcC was preferentially recovered in cellulose‐bound fractions, with the amount of bound protein increasing with MCC concentration (Fig. 1). ClcC was depleted from the supernatant across all conditions tested.

Next, the activity of the dockerin‐bearing glycoside hydrolase, ClcD, was characterised. The purified protein was incubated for 3 h with phosphoric acid‐swollen cellulose (PASC) at various concentrations, and released reducing sugars were measured by the DNS assay [20]. ClcD showed increasing specific activity with increasing PASC concentration (Fig. S6).

**Fig. 2:**
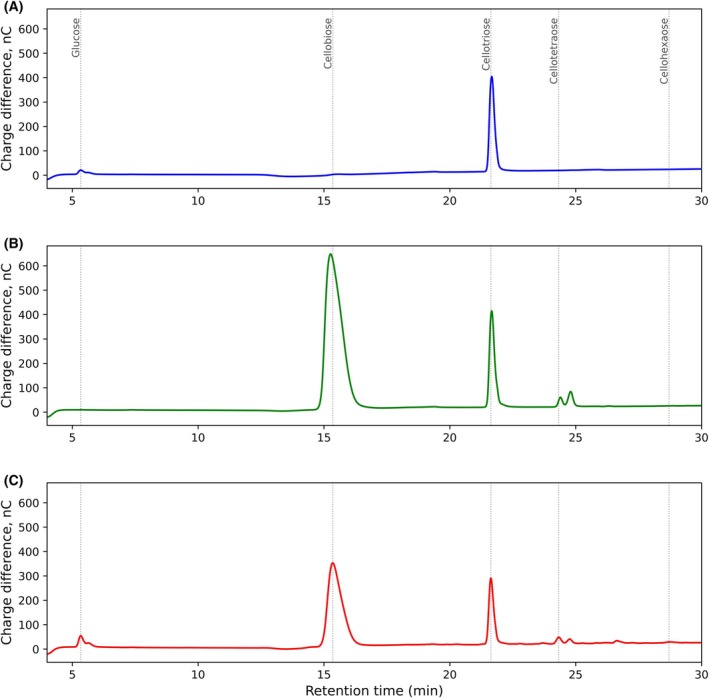
Activity of ClcD on cellodextrins. ClcD was incubated with cellotriose (A), cellotetraose (B) or cellohexaose (C), and reaction products were analysed by high‐performance anion‐exchange chromatography with pulsed amperometric detection (HPAEC‐PAD). Traces shown are representative of *n* = 3.

Next, we probed the specificity of the binding pocket of the enzyme's active site by testing activity on glucose and the relevant oligomers: cellobiose, cellotriose, cellotetraose and cellohexaose. The oligosaccharides released were analysed by HPAEC‐PAD, as shown in Fig. 2A–C. ClcD had no detectable hydrolysis products when incubated with glucose or cellobiose under our experimental conditions. It showed the strongest activity on cellotetraose, which was largely converted to cellobiose, weaker activity on cellohexaose, which was converted to cellotriose and other shorter oligosaccharides, and only a very weak preference for cellotriose (Fig. 2A–C).

The efficient turnover of cellotetraose and cellohexaose indicates that while ClcD is a relatively poor catalyst on bulk amorphous cellulose, it is well adapted to acting on shorter chain cellulose oligosaccharides. This pattern is broadly consistent with other characterised GH5‐dockerin enzymes, which efficiently hydrolyse longer cello‐oligomers to give mainly cellobiose and cellotriose, but show little activity on glucose or cellobiose [21, 22].

ClcD's preference for oligosaccharides likely reflects active site architecture documented in other GH5 enzymes, where loop structures and substrate binding pocket geometry limit accessibility to extended polymeric substrates [23]. We therefore asked whether the addition of ClcC could enhance the apparent activity of ClcD on polymeric cellulosic substrates, despite the enzyme's preference for shorter cello‐oligosaccharides.

### ClcC and ClcD form a functional complex

**Fig. 3:**
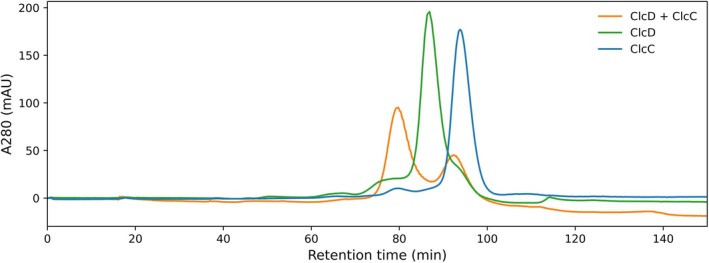
ClcC and ClcD size‐exclusion chromatography. Purified ClcC and ClcD were concentrated (~ 100×; 30 kDa molecular weight cut‐off, MWCO) and analysed by size‐exclusion chromatography on a HiLoad Superdex 200 pg 16/600 column, either individually or after mixing an equimolar ratio. A280 traces are shown for ClcD plus ClcC (orange), ClcD (green) and ClcC (blue).

The defining feature of the cellulosome is the cohesin‐dockerin interaction. To test whether the cohesin on ClcC and the dockerin on ClcD form a complex, purified proteins were incubated together at room temperature and analysed by size‐exclusion chromatography, alongside each protein analysed individually under the same conditions (Fig. 3). The mixture produced a prominent earlier eluting peak relative to either protein alone, consistent with formation of a higher apparent molecular size species. SDS/PAGE of these fractions showed two bands consistent with ClcC and ClcD (Fig. S7).

**Fig. 4:**
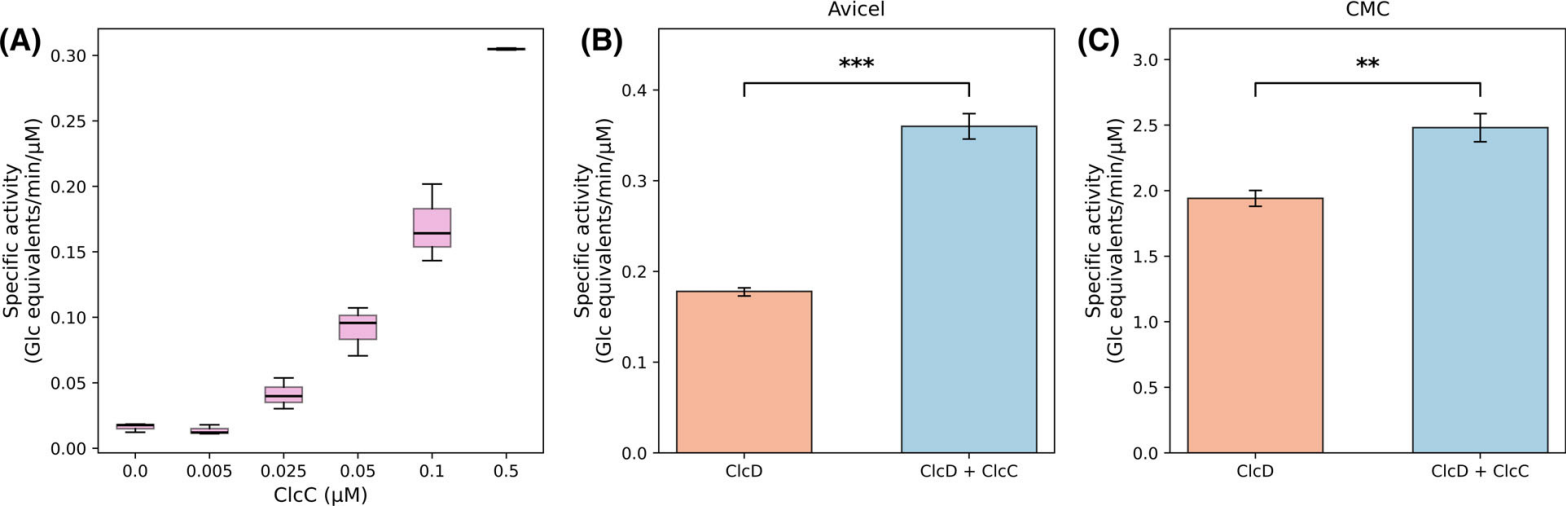
ClcC enhances ClcD cellulase activity. (A) Purified ClcD (0.05 μm) was incubated with increasing amounts of ClcC (0–0.5 μm) and activity was measured by reducing sugar release using the DNS assay. In (A), boxes span the interquartile range, the central line marks the median, and whiskers extend to the minimum and maximum values (*n* = 3 biological replicates per ClcC concentration). (B) Activity on insoluble Avicel (MCC) for ClcD alone versus ClcD plus equimolar ClcC. (C) Activity on soluble carboxymethyl cellulose (CMC) for ClcD alone versus ClcD plus equimolar ClcC. In (B) and (C), bars show mean and error bars show SD (*n* = 3 biological replicates per condition).

A second, later‐eluting peak was also observed in the mixture, eluting between the individual ClcD and ClcC peaks, consistent with incomplete conversion to the higher apparent size species under these conditions. Having confirmed ClcD and ClcC form a complex, we next sought to understand how ClcC affects the enzymatic activity of ClcD. Activity enhancement was concentration dependent, with higher ClcC concentrations yielding greater specific activity (Fig. 4A). To compare activity across substrates, reactions were set up with either ClcD alone or ClcD plus ClcC at an equimolar ratio and incubated with insoluble Avicel (Fig. 4B) or soluble carboxymethyl cellulose (CMC) (Fig. 4C). Activity was measured by the DNS assay and was higher on CMC than on Avicel. In both cases, the addition of ClcC significantly increased activity, with a larger relative enhancement on Avicel (~ 2‐fold) than on CMC (~ 1.25‐fold).

This pattern is consistent with the mechanism observed in larger cellulosomal systems, where substrate binding by CBMs increases degradation by raising the local concentration of enzyme at the cellulose surface [3, 24]. The CBM3 domain in ClcC binds strongly to cellulose, and our data show that this interaction is required to achieve higher hydrolysis rates on MCC, even in this minimal two‐component system.

*C. lentocellum's* genome encodes a number of predicted cellulose‐degradation enzymes [8] (Table 1), suggesting a strategy by which ClcD may process soluble oligosaccharide intermediates released by other cellulases. In the context of this minimal system, ClcD is therefore more likely to play a supporting role, processing soluble or partially released oligomers, rather than serving as the primary enzyme responsible for initial cellulose depolymerisation. The enhanced activity in the presence of ClcC demonstrates a functional advantage of scaffoldin‐mediated assembly even in this minimal two‐component system. Notably, ClcD was more active on soluble CMC than on insoluble Avicel, yet ClcC produced the larger enhancement on Avicel; this pattern suggests why the CBM is supplied on a separate polypeptide rather than fused to ClcD. Recruiting the CBM in trans may allow ClcD to act on soluble substrates without it, while gaining cellulose‐targeting capacity when bound to ClcC, providing a mechanistic rationale for retaining a cohesin‐dockerin interaction in such a reduced system. The single predicted promoter suggests *clcC* and *clcD* are co‐transcribed, but their ribosome‐binding sites differ in predicted strength, so ClcC is likely produced in excess of ClcD, favouring conditional supply of the CBM in trans over a fixed 1 : 1 pairing.

### Identification and structural characterisation of additional candidate ClcC‐interacting proteins

Since it was unusual that there appeared to be only one annotated dockerin‐bearing enzyme in the genome of *C. lentocellum* DSM 5427, we hypothesised that additional proteins might interact with ClcC. To identify potential partners, we performed a pull‐down experiment, as a single exploratory screen, using purified ClcC as bait against *C. lentocellum* cell‐free extract and culture supernatants.

In ClcC‐baited pull‐down experiments, ClcD was not detected. This was unexpected given the direct interaction observed between recombinant ClcC and ClcD by size‐exclusion chromatography (Fig. 3) and the functional enhancement of ClcD activity in the presence of ClcC (Fig. 4). The pull‐down was therefore interpreted as an exploratory screen for native ClcC‐associated proteins rather than as the primary evidence for the ClcC‐ClcD interaction. The absence of ClcD may reflect low native abundance under the growth conditions used. ClcD has a predicted Sec signal peptide and has a predicted translation initiation rate approximately an order of magnitude lower than that of *clcC*, which also implies that, within the shared transcript, ClcC is produced in excess of ClcD. In addition, related cellulolytic clostridia show carbon‐source‐dependent expression of cellulosomal components; for example, in *Cellulosilyticum ruminicola* H1, scaffoldin transcription is lower on cellobiose than on cellulose, and the cellulolytic complex was not recovered from high‐cellobiose cultures [25]. As the cells used here were grown on cellobiose, native ClcD may have been present below the detection threshold of the assay.

Recovered proteins were ranked by intensity rather than enrichment and treated as candidates requiring validation, hits were filtered by intensity and manually curated to remove homologues of common non‐specific binders (e.g., ribosomal proteins, EF‐Tu, chaperonins). Of the remaining candidates (Table S2), two were of particular interest: Clole_2778, an uncharacterised protein that returned the highest intensity of any non‐bait, non‐housekeeping hit, and Clole_0709, a putative cellulase. The recovery of Clole_0709, which is itself predicted to be secreted, indicates that cellulolytic proteins were present in the analysed material and that the absence of ClcD was due to neither secretion nor a general failure to recover ClcC‐associated proteins.

**Fig. 5:**
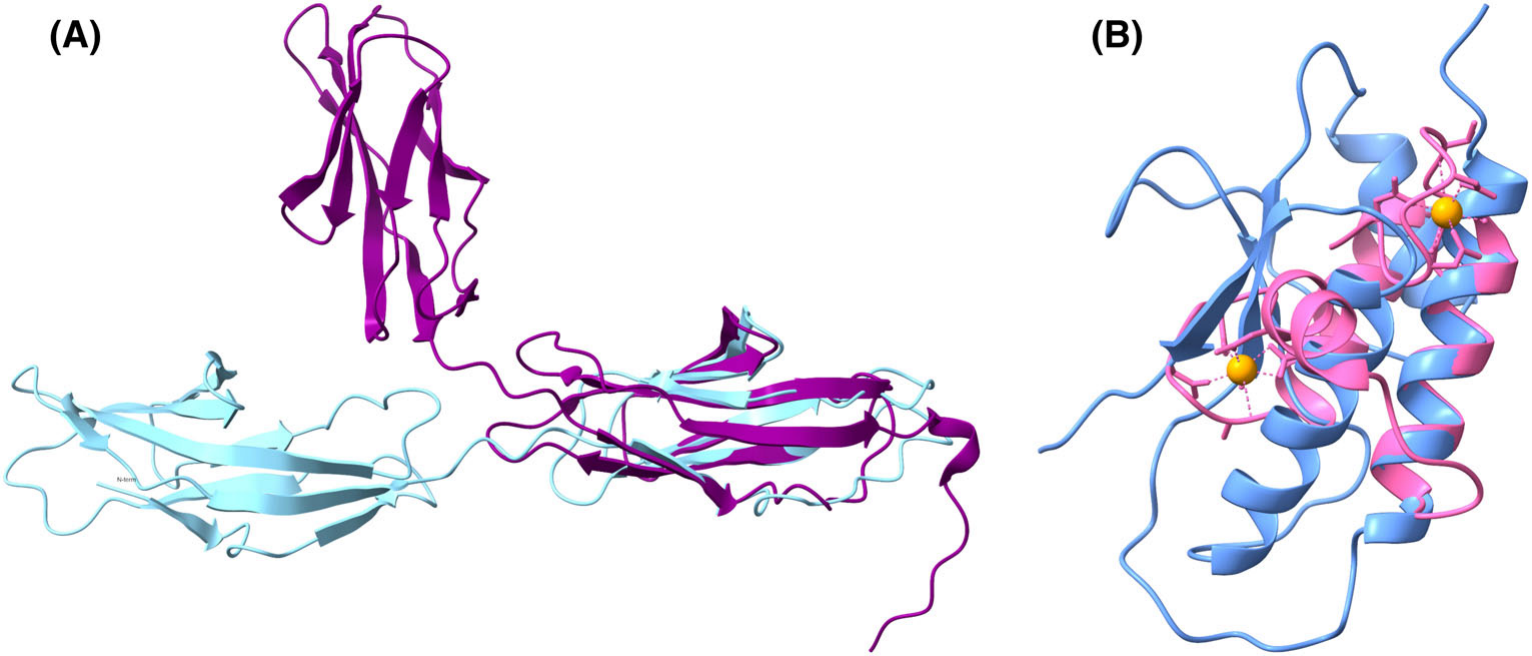
Structural comparisons of candidate ClcC interactors. alphafold2 models were searched against the Protein Data Bank (PDB) with distance‐matrix alignment (DALI) and visualised in ucsf chimerax. (A) Bacterial immunoglobulin‐like domain type VII (BIg‐7) repeat of Clole_0709 (cyan) superposed on the fibronectin type III domain of *A. thermocellum* CbhA (purple; PDB 3PDD); 38 pruned Cα pairs, root‐mean‐square deviation (RMSD) = 0.99 Å (DALI sequence‐alignment score = 242). (B) Clole_2778 model (blue) manually aligned to the canonical dockerin from *A. thermocellum* (pink; PDB 1OHZ) with chimerax matchmaker; 15 pruned Cα pairs, RMSD = 1.54 Å. Calcium ions from 1OHZ are shown as orange spheres.

A conserved‐domain BLAST search indicated that Clole_0709 encodes a putative cellulase with an enzymatic domain and a bacterial immunoglobulin‐like domain type VII (BIg‐7). Using alphafold2 (accessed via colabfold), the BIg‐7 structure was predicted and queried with a DALI structural search. The closest match was to fibronectin type III domains from CbhA of *A. thermocellus* [PDB: 3PDD] (Fig. 5A), which have been shown to aid cellulose hydrolysis by modifying the substrate surface. There is evidence that BIg‐7‐like domains can bind Ca^2+^, which is an important mediator of cohesin‐dockerin interactions [26, 27], though the functional significance of this in Clole_0709 remains unclear.

The same bioinformatic process was applied to Clole_2778, which showed no identifiable domains by conserved‐domain blast. DALI searches returned no canonical dockerin folds among the top hits. However, the alphafold2 model of Clole_2778 adopts a fold that resembles dockerins in overall topology but lacks conserved Ca^2+^‐binding motifs, with only a single, disrupted Ca^2+^‐binding loop. To illustrate this divergence, Clole_2778 was manually aligned to a canonical dockerin from *A. thermocellus* (PDB 1OHZ; Fig. 5B). While there is evidence in the literature for a diversity of structures, disrupted binding loops, and divergent calcium‐binding motifs in functional cohesin‐dockerin pairs [28, 29], the extent of deviation in Clole_2778 is consistent with a non‐canonical architecture.

To assess prototypical dockerin motif conservation at the primary sequence level, Clole_0709 (BIg‐7 repeats; residues 696–760 and 798–869) and Clole_2778 were aligned with representative canonical dockerin domains (CelCca, XynY [PDB: 1OHZ], CelS [PDB: 1DAQ]) using clustal omega and visualised with ESPript [30] (Fig. S8). In canonical type I dockerins, cohesin binding depends on Ca^2+^‐mediated stabilisation of EF‐hand‐like loop–helix motifs, hydrophobic complementarity between the dockerin and cohesin surfaces, and a network of hydrogen bonds reinforcing the interface. Ca^2+^ is typically coordinated by two 12‐residue EF‐hand‐like loops, and a conserved Ser/Thr pair at loop positions 10–11 often contributes to interface hydrogen bonding [17, 31].

For Clole_0709, only the BIg‐7 region was analysed, as the GH9 catalytic core and CBM3 modules are not expected to mediate cohesin binding. This region showed the best, though still weak, resemblance to the dockerin secondary‐structure pattern, but lacked the hallmark EF‐hand Ca^2+^ binding loops and the conserved Ser/Thr pair (Fig. S8).

Similarly, in Clole_2778, multiple deviations from the standard architecture are observed (Fig. S8). The first Ca^2+^‐binding loop is absent; the conserved Ser/Thr pair is replaced by a non‐polar Met/Ile, disfavoured for hydrogen bonding; and the second loop is disrupted, with only the aspartate at position 3 conserved. Additionally, a 27‐residue insertion containing a β‐strand and an α‐helix interrupts the expected helix–loop–helix architecture.

Clole_2778 was recovered with high intensity in ClcC‐baited pull‐downs (1.27 × 10^9^; Table S2), among the highest of all intensity hits. Homology searches returned no close homologues outside *C. lentocellum*, suggesting that this protein may be specific to *C. lentocellum*. The absence of any identifiable catalytic domain distinguishes it from typical cellulosomal components and may point to a structural or accessory role within such a complex.

These results suggest that ClcC may interact with proteins beyond canonical dockerin‐bearing enzymes, potentially through alternative binding mechanisms. To explore this further, alphafold‐multimer was used to model potential ClcC‐Clole_2778 and ClcC‐Clole_0709 complexes. alphafold‐multimer modelling of Clole_0709 with ClcC yielded low inter‐chain confidence scores across all domain combinations tested and was not pursued further.

The top‐ranked ClcC‐Clole_2778 model returned high confidence scores (ipTM ~ 0.85, interface pLDDT ~ 90%), with the predicted interface differing from canonical cohesin‐dockerin binding modes (Fig. S4). These computational predictions, together with the pull‐down data, suggest Clole_2778 as a candidate non‐canonical ClcC‐interacting partner. We therefore present an alternative, non‐canonical binding mode as a hypothesis requiring experimental validation rather than as an established mechanism.

## Conclusions

In the genome of *C. lentocellum*, we have identified and characterised a functional cohesin‐dockerin system built from a single cohesin‐bearing protein and a single dockerin‐bearing enzyme. This system performs the core cellulosomal function, a cohesin‐dockerin interaction that recruits a catalytic module to a cellulose‐targeting scaffold and enhances activity on insoluble substrate.

However, canonical cellulosomes are normally enormous, branching protein complexes defined by a scaffoldin carrying two or more cohesins together with at least one dockerin‐fused glycoside hydrolase [15]. ClcC, the only cohesin‐bearing protein in the genome, carries a single cohesin domain, so this reduced system does not meet that definition. Yet it is equally distinct from the wider population of non‐cellulosomal cohesin‐ and dockerin‐bearing proteins, whose parent proteins are predominantly non‐catalytic [32], whereas here the dockerin is fused to a functional cellulase. Its components, a CBM‐bearing cohesin protein and a dockerin‐fused GH5, are unambiguously cellulosomal, yet the system as a whole sits between the categories conventionally used to describe cohesin‐dockerin proteins. We therefore argue that it is most usefully understood as a minimal cellulosome system.

The *C. lentocellum* system shows that a single cohesin paired with a single dockerin‐bearing enzyme can still deliver functional advantages comparable to those of larger systems, namely enhanced activity on insoluble substrates and the potential for combinatorial assembly. At the same time, the identification of a candidate non‐canonical interaction partner raises the possibility, to be tested, that cohesin‐dockerin recognition is more structurally diverse than previously appreciated.

## Materials and methods

### Gene identification and phylogenetic analysis

Genes *clcC* and *clcD* were identified by PSI‐BLAST [33] searches of a pre‐defined library of strains of the DSMZ GEBA collection of microorganisms. We hold a copy of this collection in our strain stocks. Query sequences used were alignments of the Pfam cohesin domain (PF10345) group. This resulted in hits of non‐novel domains from organisms in the collection such as *Acetivibrio thermocellus* (previously *Clostridium thermocellum*).

*C. lentocellum* cohesin and dockerin sequences identified in this search were aligned with canonical Pfam sequences for these domains using clustalw (Fig. S1). Based on these alignments, phylogenetic trees were generated from curated representative sequence sets with incomplete sequences removed, comprising 79 dockerin sequences (Fig. S2) and 78 cohesin sequences (Fig. S3).

CAZyme searches were performed on the *Cellulosilyticum lentocellum* DSM 5427 proteome (GenBank CP002582.1) using dbCAN3 [34] with integrated (a) HMMER against the dbCAN HMM database, (b) DIAMOND against the CAZy database, and (c) dbCAN_sub. Annotations found by at least two methods were retained. Signal peptides were predicted with SignalP 6.0 [35], and proteins lacking a signal peptide were classified as cytoplasmic.

### Cultivation of *C. lentocellum*

*C. lentocellum* (obtained from the DSMZ) was cultured for ~ 2 weeks in peptone cellulose solution B (PCSb) consisting of 2.0 g·L^−1^ peptone, 0.882 g·L^−1^ CaCl_2_·2H_2_O, 1.0 g·L^−1^ yeast extract and 5.0 g·L^−1^ NaCl, at 30 °C under anaerobic conditions without shaking. Genomic DNA was prepared from the resulting culture using the GenElute Microbial DNA Extraction Kit (Sigma‐Aldrich, Gillingham, Dorset, UK).

### Cloning of *clcC* and *clcD*

Primers were generated which would amplify and add BamHI and HindIII sites to the 5′ and 3′ ends of both *clcD* and *clcC*. After amplification of the genes with these primers from genomic DNA, the fragments were digested with appropriate restriction enzymes (NEB) and ligated using T4 DNA ligase (NEB) into pET28a for *clcD* and pQE80L for *clcC* (yielding pQEClSca). After unsuccessful attempts to purify ClcD protein, a second plasmid was produced by deletion of the putative Sec signal peptide (residues 1–27) from the gene using the Q5^®^ Site‐Directed Mutagenesis Kit (NEB) to generate pETClGHdsec, which expresses ClcD with an uncleaved N‐terminal His tag. All primer sequences are available in the Table S1.

### Protein production and purification

ClcD was produced in BL21(DE3) *E. coli* by transformation with pETClGHdsec and cultured in autoinduction medium (Formedium; 10 g·L^−1^ tryptone, 5 g·L^−1^ yeast extract, 3.3 g·L^−1^ ammonium sulfate, 6.8 g·L^−1^ potassium dihydrogen phosphate, 7.1 g·L^−1^ disodium hydrogen phosphate, 0.5 g·L^−1^ glucose, 2.0 g·L^−1^ lactose, 0.15 g·L^−1^ magnesium sulfate) overnight at 30 °C with shaking at 200 rpm. ClcC was produced in BL21(DE3) transformed with pQEClSca and cultured at 20 °C for 48 h at 200 rpm in the same autoinduction medium. Typically, 500 mL cultures were used and then aliquoted into 50 mL portions; cells were harvested and stored as pellets at −20 °C until lysis.

For purification, cell pellets were resuspended in 10 mL calcium dilution and incubation (CDI) buffer per g wet cells (50 mm Tris pH 8.0, 50 mm NaCl, 12 mm CaCl_2_). Suspensions were sonicated (Branson Digital Sonifier, Danbury, CT, USA) and clarified at 25 000× ***g*** to remove insoluble material. The supernatant (cell‐free extract) was applied to 5 mL TALON resin (Takara Bio USA, San Jose, CA, USA) equilibrated in CDI buffer, washed with CDI buffer and then CDI + 10 mm imidazole and eluted with CDI + 100 mm imidazole. Proteins were concentrated and buffer‐exchanged using Millipore centrifugal filter units before use.

ClcC and ClcD were purified in parallel, and each chromatographed individually on a HiLoad 16/600 Superdex 200 pg column (Cytiva, Marlborough, MA, USA) pre‐equilibrated in CDI buffer. For the complex run, the two proteins were mixed at an equimolar ratio, concentrated approximately 100‐fold with a 30 000 MWCO centrifugal filter, and applied to the same column under identical conditions. Fractions collected at selected peaks were also analysed by SDS/PAGE.

### Avicel pull‐downs

Purified ClcC was incubated with microcrystalline cellulose (MCC; Avicel PH‐101, Sigma‐Aldrich) in CDI buffer for 3 h at 4 °C with rotary shaking, across an MCC concentration series. The MCC was pelleted by centrifugation, washed with CDI buffer, and the bound (pellet) and unbound (supernatant) fractions were prepared for SDS/PAGE by boiling in Laemmli sample buffer for 10 min.

### Enzymological methods

Purified ClcD was diluted in CDI buffer to 40 μm and incubated at 30 °C with 0.05% (m/v) of the designated substrate prepared in the same buffer. For apparent kinetic measurements on phosphoric acid‐swollen cellulose (PASC), reactions contained 0–400 mg·mL^−1^ PASC and were incubated for 3 h. Reducing sugars were quantified by the 3,5‐dinitrosalicylic acid (DNS) assay [20] using cellobiose standards; substrate‐only blanks were subtracted. Where indicated, soluble substrates (glucose and cello‐oligosaccharides, DP2‐6) were analysed by HPAEC‐PAD on a Dionex ICS‐5000 with a CarboPac PA10 column, according to Muñoz *et al*. [36].

### Baited cohesin pull‐downs

Cell‐free extract (CFE) from BL21(DE3) *E. coli* expressing pQEClSca (ClcC) was applied to 5 mL TALON resin pre‐equilibrated with CDI buffer (50 mm Tris pH 8.0, 50 mm NaCl, 12 mm CaCl_2_) to capture His‐tagged ClcC. The resin was washed with CDI, then with CDI + 10 mm imidazole. Calcium‐containing CDI buffer was used throughout ClcC immobilisation, binding and washing.

*C. lentocellum* was grown in 50 mL PCSb supplemented with cellobiose overnight at 30 °C and harvested; CFE and culture supernatants were prepared as described above. This *C. lentocellum* CFE and supernatant were incubated with the ClcC‐charged resin, followed by washes with CDI. ClcC and any interacting partners were eluted with CDI + 100 mm imidazole. Eluates were concentrated ~ 10‐fold using Millipore centrifugal filter units (MilliporeSigma, Burlington, MA, USA) and analysed by SDS/PAGE.

### Protein–protein interaction modelling

Single‐chain models of ClcC, Clole_2778 and Clole_0709 were generated with alphafold2 using colabfold v1.5.5 (default MMseqs2 MSA settings; templates off unless stated) [37]. Putative cohesin‐dockerin complexes were then modelled with alphafold‐multimer v2.3.2 (colabfold) by pairing the ClcC cohesin segment (residues 290–414) with either Clole_0709 (696–869) or Clole_2778 (1–107). For each pairing, we enabled extra chain‐pair scoring, used 24 recycles and generated 24 models (distinct random seeds) [38, 39]. Models were ranked by inter‐chain pTM (ipTM), and the top‐ranked ipTM model for each pairing was carried forward for analysis; per‐residue pLDDT and PAE plots are provided in the Supporting Information (Fig. S4).

## Conflict of interest

The authors declare no conflict of interest.

## Author contributions

JA contributed to conceptualization, investigation, formal analysis, methodology, writing—original draft, editing, reviewing. AA‐M contributed to writing—reviewing and editing, bioinformatics‐analysis, methodology. GB contributed to conceptualization, validation, supervision, reviewing and editing, funding acquisition, project administration.

## Supporting information


**Fig. S1.** *C. lentocellum* cohesin and dockerin domain alignments with close relatives.
**Fig. S2.** Dockerin tree.
**Fig. S3.** Cohesin tree.
**Fig. S4.** alphafold‐Multimer confidence and model for candidate ClcC partners.
**Fig. S5.** The *Cellulosilyticum lentocellum* genome encodes an uncharacterised operon that contains novel cohesin and dockerin domains.
**Fig. S6.** Activity of ClcD on phosphoric acid‐swollen cellulose (PASC).
**Fig. S7.** SDS/PAGE of size‐exclusion chromatography fractions across the ClcC‐ClcD complex peak.
**Fig. S8.** Clole_2778 and the Clole_0709 BIg‐7 domain deviate from the canonical type I dockerin fold.
**Table S1.** Primer list for cloning of *clcD* and *clcC*.
**Table S2.** Proteins identified in the ClcC‐baited pull‐down.

## Data Availability

alphafold2 and alphafold‐multimer models, together with the input sequences and colabfold run parameters, are available from zenodo (doi: 10.5281/zenodo.21671695). The processed mass spectrometry proteomics data have been deposited to Zenodo (doi: 10.5281/zenodo.21822190). All other data are available from the authors upon reasonable request.
