# Supplementary material for: A minimal cellulosome‐like system in Cellulosilyticum lentocellum
Source: FEBS Open Bio. 2026 Aug 19:10.1002/2211-5463.70330. Online ahead of print. doi: 10.1002/2211-5463.70330 (PMC13487519; doi:10.1002/2211-5463.70330)
Supplement: Supplementary file 1 [file FEB4-9999-0-s001.docx]

**Supplementary Figure 1.** *C. lentocellum* cohesin and dockerin domain alignments with close relatives. BLASTp was used with the *C. lentocellum* cohesin and dockerin sequences as queries against the NCBI nr database. Alignments were then produced using the Smith-Waterman method and visualised using ESPript [35]. (A) ClcD dockerin domain (449-518) aligned with the *Acetivibrio thermocellus* XynB dockerin (identity 57%; accession: WP_407652294.1) and the *Acetivibrio clariflavus* glycoside hydrolase family 9 (GH9) dockerin domain (identity 59.65%; accession: WP_313563123.1). (B) ClcC cohesin domain (287-419) aligned with that of *Cellulosilyticum* sp. WCF-2 and cohesin domain of *Iocasia fonsfrigidae* (identity 35.16%; accession: WP_230868803.1).

**Supplementary Figure 2.** Dockerin tree. Phylogenetic tree of representative dockerin domain sequences (n = 79) generated from a curated sequence set with incomplete sequences removed. iTOL was used to visualise the phylogenetic trees. *C. lentocellum* sequences are highlighted in red.

**Supplementary Figure 3.** Cohesin tree. Phylogenetic tree of representative cohesin domain sequences (n = 78) generated from a curated sequence set with incomplete sequences removed. iTOL was used to visualise the phylogenetic trees. The *C. lentocellum* cohesin (ClcC) is highlighted in red.

**Supplementary Figure 4.** AlphaFold-Multimer confidence and model for candidate ClcC partners. (A) Chain-pair confidence matrix for the rank-1 ClcC-Clole_2778 complex (ipTM = 0.84). (B) Chain-pair confidence matrix for the rank-1 ClcC-Clole_0709 BIg-7 fragment (residues 696-869) complex (ipTM = 0.39). (C) Rank-1 AlphaFold-Multimer model of ClcC (pink surface/ribbon) bound to the dockerin-like domain of Clole_2778 (blue surface/ribbon). Residues at the predicted interface (≤ 2 Å inter-chain distance) are shown in green.

**Supplementary Figure 5.** The *Cellulosilyticum lentocellum* genome encodes an uncharacterised operon that contains novel cohesin and dockerin domains. By position-specific iterated basic local alignment search tool (PSI-BLAST) search of genomes in the Genomic Encyclopedia of Bacteria and Archaea (GEBA) collection of microorganisms (German Collection of Microorganisms and Cell Cultures, DSMZ), a novel pair of cohesin and dockerin domains was identified. (A) Operon structure of *clcC* and *clcD*. A single promoter (black arrow) was predicted upstream of *clcC* using BPROM. The two half-ovals mark ribosome-binding sites predicted by the RBS Calculator, labelled with the relative predicted translation-initiation rates in arbitrary units. Putative domains were annotated using the BLAST conserved domain search and the EMBL SMART tools. SMART cartoon output is shown here for ClcC (B) and ClcD (C).

**Supplementary Figure 6.** Activity of ClcD on phosphoric acid-swollen cellulose (PASC). ClcD was incubated with PASC (0-400 mg/mL) in CDI buffer at 30 °C for 3 h, and reducing sugars were quantified by the 3,5-dinitrosalicylic acid (DNS) assay.

**Supplementary Figure 7. Sodium dodecyl sulfate polyacrylamide gel electrophoresis (SDS-PAGE) of size exclusion chromatography fractions across the ClcC-ClcD complex peak.** Fractions across the earlier-eluting peak from the mixed ClcC and ClcD run shown in Figure 3 were analysed by SDS-PAGE. Two bands were present at apparent masses consistent with ClcC and ClcD.

**Supplementary Figure 8.** (A) Multiple-sequence alignment of Clole_2778 with dockerin domains from *A. thermocellus* (XynY, CelS), *A. cellulolyticus* (CelCca) and *C. lentocellum* (ClcD), together with the Clole_2778 AlphaFold2 model annotated to highlight deviations from the canonical type I dockerin fold. Gold: truncated first Ca²⁺-binding loop (no GDxNxD motif). Red: Met 1 / Ile 2 replacing the conserved Ser/Thr hydrogen-bond pair. Pink: disrupted second Ca²⁺-binding loop (VTGEDGGTANLDS); the only retained Asp is shown in green. Purple: 27-residue β-strand/α-helix insertion interrupting the helix-loop-helix architecture. Yellow: position of the second Ser/Thr pair. (B) Multiple-sequence alignment of the Clole_0709 BIg-7 (bacterial immunoglobulin-like domain type VII) domain (residues 696-869) with the same canonical dockerins and its AlphaFold2 model. The domain forms an immunoglobulin-like β-sandwich (β1-β15) rather than the α-helical dockerin fold. Lime green marks residues structurally equivalent to the first canonical Ca²⁺-binding loop; GDxNxD and Ser/Thr motifs are absent, and no EF-hand loops or α-helical dockerin motifs are present. Visualised using ESPript [35].

| **Primer name** | **Sequence (5′→3′)** | **Restriction site** | **Purpose** |
| --- | --- | --- | --- |
| JA-ClScaF | GCATCACCATCACCATCAC**GGATCC**GTAAAACTGGAATATTGTAATACG | BamHI | Amplification of *clcC* |
| JA-ClScaR | GATAACGGTTCTATTTAAAGTGA**AAGCTT**AATTAGCTGAGCTTGGACT | HindIII | Amplification of *clcC* |
| JA-ClGH5a-F | GCATCACCATCACCATCAC**GGATCC**AAAATAGGGAAGAAAGTAACAAG | BamHI | Amplification of *clcD* |
| JA-ClGH5a-R | GAAATAGTGATAGCCTGCAATA**AAGCTT**AATTAGCTGAGCTTGGACT | HindIII | Amplification of *clcD* |
| JA-ClGH5-secdelF | CATATGGCTGCCGCGCGG | - | Site-directed mutagenesis of *clcD* (signal peptide deletion) |
| JA-ClGH5-secdelR | GCTGCTACAGCAAAATCAGATGATGATTGGT | - | Site-directed mutagenesis of *clcD* (signal peptide deletion) |

**Supplementary Table 1. Primer list for cloning of [clcD] and [clcC].** The restriction sites (NdeI, XhoI, BamHI, HindIII) are shown in bold

| **Protein name** | **Gene name(s)** | **Locus ID** | **UniProt ID** | **Intensity** | **Peptides** | **Unique peptides** | **Sequence coverage [%]** |
| --- | --- | --- | --- | --- | --- | --- | --- |
| ClcC (Bait) | *clcC* | Clole_2599 | F2JID6 | 1.28E+11 | 35 | 35 | 90.9 |
| Ribosomal RNA small subunit methyltransferase A (EC 2.1.1.182) | *rsmA, ksgA* | Clole_0654 | F2JN46 | 2.59E+09 | 1 | 1 | 2.8 |
| **Uncharacterised protein** |  | **Clole_2778** | **F2JK62** | **1.27E+09** | **1** | **1** | **16.8** |
| Elongation factor Tu (EF-Tu) | *tuf* | Clole_3548 | F2JT13 | 3.88E+08 | 5 | 5 | 15.4 |
| Cadmium-translocating P-type ATPase (EC 3.6.3.3) |  | Clole_3672 | F2JH15 | 2.69E+08 | 1 | 1 | 2.4 |
| 50S ribosomal protein L6 | *rplF* | Clole_3531 | F2JSH7 | 2.03E+08 | 1 | 1 | 14.0 |
| **Cellulase (EC 3.2.1.4)** |  | **Clole_0709** | **F2JNU4** | **1.79E+08** | **1** | **1** | **3.8** |
| Integral membrane sensor signal transduction histidine kinase |  | Clole_3138 | F2JP94 | 5.36E+07 | 1 | 1 | 2.8 |
| Histidine kinase (EC 2.7.13.3) |  | Clole_3860 | F2JJE5 | 2.62E+07 | 1 | 1 | 5.1 |
| Transcriptional repressor, CopY family |  | Clole_4056 | F2JL89 | 1.80E+07 | 1 | 1 | 12.5 |
| Transcription termination factor Rho (ATP-dependent helicase Rho) | *rho* | Clole_1626 | F2JKU8 | 1.41E+07 | 2 | 2 | 4.6 |
| 60 kDa chaperonin (GroEL protein) (Cpn60) | *groL, groEL* | Clole_3332 | F2JR41 | 1.39E+07 | 2 | 2 | 6.2 |
| Phosphoglycerate kinase (EC 2.7.2.3) | *pgk* | Clole_2834 | F2JKZ2 | 1.23E+07 | 1 | 1 | 1.7 |
| ATP-dependent transcriptional regulator, MalT-like, LuxR family |  | Clole_3186 | F2JPU5 | 1.20E+07 | 1 | 1 | 2.5 |
| Signal recognition particle protein (Fifty-four homolog) | *ffh* | Clole_2306 | F2JS77 | 1.13E+07 | 1 | 1 | 2.9 |

**Supplementary Table 2.** Proteins identified in the ClcC baited pull-down. Bands were excised, subjected to in-gel trypsin digestion, and analysed by liquid chromatography tandem mass spectrometry (LC-MS/MS). Peptides were searched with MaxQuant against the UniProt predicted proteome for *C. lentocellum* (UP000008467). Hits with intensities <10⁶ were discarded. Peptide counts, unique peptides, and sequence coverage are shown for each protein. Identifications are listed in order of intensity, with entries of particular interest shown in bold.
